# Cardiac troponin I and the risk of cardiovascular or non-cardiovascular death in patients visiting the emergency department

**DOI:** 10.1038/s41598-021-96951-y

**Published:** 2021-08-31

**Authors:** Jong Eun Park, Minseok Song, Taerim Kim, Gun Tak Lee, Sung Yeon Hwang, Hee Yoon, Won Chul Cha, Tae Gun Shin, Min Sub Sim, Ik Joon Jo, Seung-Hwa Lee, Hyung-Doo Park, Jin-Ho Choi

**Affiliations:** 1 Department of Emergency Medicine, Samsung Medical Center, Sungkyunkwan University School of Medicine, 81, Irwon-ro, Gangnam-gu, Seoul, 06351 Republic of Korea; 2 Department of Medicine, Samsung Medical Center, Sungkyunkwan University School of Medicine, Seoul, Republic of Korea; 3 Department of Laboratory Medicine and Genetics, Samsung Medical Center, Sungkyunkwan University School of Medicine, Seoul, Republic of Korea

**Keywords:** Prognostic markers, Biomarkers

## Abstract

The prognostic implication of cardiac troponin I (cTnI) values for the determination of the magnitude or duration of cause-specific death risk is limited. We included consecutive patients with maximal cTnI values within 24 h of their emergency department visits. Multivariate analyses using variables selected by the Bayesian information criterion were performed to investigate the impact of cTnI on the event rate, time-dependent risk, and dose-dependent risk of cardiovascular or non-cardiovascular death within 360 days. There were 5472 (14.9%) all-cause deaths including 881 (2.4%) cardiovascular deaths and 4591 (12.5%) non-cardiovascular deaths. In patients with positive cTnI, defined as the ≥ 99th percentile of the upper normal limit, the cumulative risk of cardiac and non-cardiac death was 4.4- and 1.4-fold higher, respectively, than that of negative cTnI, respectively. In the competing risk analysis, positive cTnI was linked to 2.4- and 1.2-fold higher risks of cardiovascular and non-cardiovascular death, respectively. The cTnI value showed a positive relationship with the risk of both cardiovascular and non-cardiovascular deaths. In the time-dependent risk analysis, the excess risk of cardiovascular death was mostly evident in the first few weeks. Higher cTnI value was associated with an increased risk of both cardiovascular and non-cardiovascular death, especially which was in the early period.

## Introduction

Cardiac troponin is highly specific to myocardial injury, and is the gold standard for the screening of potential acute coronary syndrome or rapid diagnosis of acute myocardial infarction in the emergency department^1,2^. Increased penetration of cardiac troponin tests led to a considerable increase in the number of patients with elevated cardiac troponin levels of uncertain clinical significance^3^. It is often difficult how to choose the best treatment strategy or risk stratification for these patients.

Cardiac troponin levels are reported as a continuous scale that may have a positive relation with excess risk. In clinical practice, the results are often interpreted as binary positive or negative results, based on the 99th percentile upper limit of normal (ULN). However, this method can sometimes be too simple and can ignore the information provided by a continuous marker of myocardial injury^1,4–6^. In addition, elevation of cardiac troponins in non-cardiac major diseases including sepsis, stroke, or critically ill status is not uncommon. Thus, diagnosis and decision-making are often challenging in clinical practice^3^.

It would be reasonable that the magnitude and the duration of excess risk are key for the interpreting the result of cardiac troponin test, which are helpful for risk stratification and prioritizing the treatment target. However, data regarding the relationship among the whole range of cardiac troponin levels and the magnitude or duration of cause-specific death risk are limited. In this study, we investigated the magnitude, temporal, and causal implications of cardiac troponin levels on the risk of cardiovascular or non-cardiovascular death among unselected patients visiting the emergency department.

## Materials and methods

### Study population

This study was conducted retrospectively using data retrieved from the Darwin-C, a dedicated clinical data warehouse at the Samsung Medical Center. The study population was extracted from a clinical data warehouse and not from study participants. The Samsung Medical Center Institutional Review Board approved this study and determined that this study complied with the Helsinki Declaration, and did not require informed consent given the use of anonymized database and the reporting of aggregated results.

Data were retrieved from patients with age ≥ 18 years old who visited the emergency department of Samsung Medical Center and who underwent cardiac troponin I (cTnI) testing within 24 h, between January 2007 and May 2016.

Patients who underwent resuscitation, mechanical circulatory support, or diagnosis of ST-elevation myocardial infarction were excluded. For those with more than one visit during the study period, only the first visit was eligible to be used in the study.

### Definition and outcomes

All tests were performed using Siemens ADVIA Centaur XP analyzer (Munich, Germany), which has the lowest cTnI analytical sensitivity of 0.006 ng/ml, upper limit normal (ULN) with 99th percentile level of 0.040 ng/ml, and coefficient of variation < 10% at 0.030 ng/ml^7^. The maximal cTnI value within 24 h of the emergency department visit was used for analysis.

The study index day was the day of the patient’s visit to the emergency department. Life status and cause of death were validated by the National Statistics of Korea. No patient was lost to follow-up with respect to death. The main cause of death was determined by the death codes in the 10th revision of the International Statistical Classification of Diseases and Related Health Problems (ICD-10). Primary outcome of interest was 360-day cardiac or non-cardiac deaths.

### Statistical analysis

Categorical and continuous variables are shown as medians with interquartile ranges and numbers with percentages, respectively. Variables among groups were compared using Chi-square or *t* tests as appropriate.

Although cTnI value is supposed to be proportional to the magnitude of risk, it is not clear whether the relationship between cTnI and mortality is valid for the whole range of cTnI, nor how long the duration of excess risk is likely to be. Therefore, the relationship between cTnI levels and the risk of death was investigated in three ways. First, cTnI value was dichotomized into positive (≥ × 1 ULN, ≥ 0.040 ng/ml) and negative cTnI (< × 1 ULN, < 0.040 ng/ml). Second, cTnI value was standardized as a ratio to the ULN value. Then it was logarithmically converted into the following intervals consisted of − 0.75, − 0.5, 0, 0.5, 1.0, 1.5, 2.0, 2.5, and 3.0; these correspond to × 0.18, × 0.32, × 1, × 3.16, × 10, × 31.6, × 100, × 316, × 1000 ULN, respectively. Third, cTnI value was treated as a continuous variable.

Most clinical conditions accompanying cTnI elevation are acute illness, and the assumption of constant proportional risk over the entire follow-up period may not be valid. Therefore, in addition to conventional Cox proportional hazard models, the impact of positive or negative cTnI on the risk of death after the early high-risk phase was assessed by landmark analysis at 30 days. Results are presented as hazard ratio (HR) and 95% confidence intervals (CI).

The impact of cTnI on the temporal change of hazard was assessed by time-dependent flexible parametric survival models for correlated time‐to‐event data^8,9^. Hence cardiac and non-cardiac death were mutually exclusive events, the competing risk of cardiovascular or non-cardiovascular death was assessed using the proportional subdistribution hazard regression model described by Fine and Gray^10^. The relationship between cTnI and risk of death was assessed using a restricted penalized spline model.

For the multivariate-adjusted analysis, the following clinical variables were included based on the lowest Bayesian information criterion (BIC) for all-cause death: visiting year, age, sex, hypertension, history of coronary artery disease, respiratory disease, hepatic disease, cancer, resuscitation, endotracheal intubation, use of vasopressors, chest pain, systolic blood pressure, diastolic blood pressure, heart rate, hemoglobin, white blood cell count, c-reactive protein, and the frequency of cTnI test administration during the initial 24 h period following the emergency department visit.

For the sensitivity analysis, all binary clinical variables except laboratory test data were used: age ≥ 65 years, sex, smoking, diabetes, hypertension, chronic kidney disease, lung disease, liver disease, prior history of stroke, prior history of coronary artery disease, prior history of myocardial infarction, cancer, resuscitation, endotracheal intubation, use of vasopressors, chest pain, hypotension defined by mean arterial pressure < 70 mmHg, and tachycardia defined as ≥ 100/min.

R version 4.0 (R Foundation for Statistical Computing) was used for statistical analyses. Statistical significance was set at p < 0.05.

## Results

### Patients and baseline clinical characteristics

**Figure 1:**
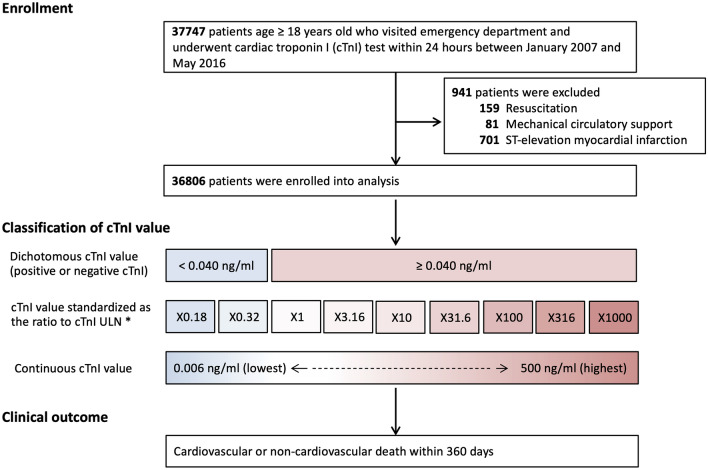
Study flow. *cTnI* cardiac troponin I, *ULN* upper limit of normal. *cTnI values were standardized as ratios to the ULN of cTnI. The ratios were logarithmically converted and categorized into intervals of − 0.75, − 0.5, 0, 0.5, 1.0, 1.5, 2.0, 2.5, 3.0, which correspond to × 0.18, × 0.32, × 1, × 3.16, × 10, × 31.6, × 100, × 316, × 1000 ULN, respectively.

A total of 36,806 patients were included (Fig. 1). The median age was 61 years (interquartile range 49–72 years) and 20,382 (55.4%) of patients were male. Compared to 30,713 (83.4%) patients with negative cTnI, 6093 (16.6%) patients with positive cTnI were older, more likely to be men, and had a higher frequency of diabetes, hypertension, and other comorbidities. Patients with positive cTnI also showed lower blood pressure and higher heart rate, and underwent more treatment for critically ill diseases represented by endotracheal intubation and use of vasopressors (Table 1).

**Table 1:** Clinical characteristics according to positive or negative cTnI.

	Negative cTnI (N = 30,713)	Positive cTnI (N = 6093)	p-value
Age (years)	59 (48–70)	67 (56–76)	< 0.001
Age ≥ 65 years	11,862 (38.6)	3457 (56.7)	< 0.001
Male sex	16,726 (54.5)	3656 (60.0)	< 0.001
Diabetes	3487 (11.4)	1222 (20.1)	< 0.001
Hypertension	8632 (28.1)	2661 (43.7)	< 0.001
Chronic kidney disease	1216 (4.0)	641 (10.5)	< 0.001
Smoking	2194 (7.1)	778 (12.8)	< 0.001
History of stroke	1081 (3.5)	392 (6.4)	< 0.001
History of coronary artery disease	624 (2.0)	305 (5.0)	< 0.001
History of myocardial infarction	130 (0.4)	122 (2.0)	< 0.001
Respiratory disease	2168 (7.1)	517 (8.5)	0.001
Hepatic disease	1386 (4.5)	382 (6.3)	< 0.001
Cancer	7711 (25.1)	1545 (25.4)	0.47
Endotracheal intubation	418 (1.4)	515 (8.5)	< 0.001
Use of vasopressors	1223 (4.0)	1105 (18.1)	< 0.001
**Symptom< 0.001**			
Chest pain	4871 (15.9)	1288 (21.1)	
Dyspnea	4589 (14.9)	1505 (24.7)	
Dysuria or urologic symptom	99 (0.3)	26 (0.4)	
Febrile symptom	1711 (5.6)	446 (7.3)	
General weakness	481 (1.6)	129 (2.1)	
Gastrointestinal symptom	3846 (12.5)	437 (7.2)	
Miscellaneous	120 (0.4)	1 (0.0)	
Neurologic symptom	2072 (6.7)	532 (8.7)	
Non-cardiac chest pain	303 (1.0)	30 (0.5)	
Not classified	8450 (27.5)	1313 (21.5)	
Syncope or palpitation	3673 (12.0)	346 (5.7)	
Trauma	498 (1.6)	40 (0.7)	
Systolic blood pressure (mmHg)	131 (114–149)	127 (106–148)	< 0.001
Diastolic blood pressure (mmHg)	80 (70–90)	76 (65–88)	< 0.001
Heart rate (/min)	84 (72–99)	91 (76–111)	< 0.001
Mean arterial pressure ≤ 70 mmHg	1811 (6.0)	758 (12.6)	< 0.001
Heart rate > 100/min	7463 (24.6)	2283 (38.0)	< 0.001
White blood cell (× 10^3^/mm^3^)	7.6 (5.9–10.1)	9.0 (6.7–12.7)	< 0.001
Hemoglobin (g/dL)	13.2 (11.6–14.6)	12.4 (10.6–14.1)	< 0.001
Platelet (× 10^3^/mm^3^)	213 (168–260)	196 (145–250)	< 0.001
Creatinine (mg/dL)	0.80 (0.65–0.99)	1.02 (0.78–1.59)	< 0.001
c-reactive protein (mg/dL)	0.22 (0.05–2.02)	1.24 (0.18–7.42)	< 0.001
Frequency of cTnI test within 24 h	1 (1–1)	1 (1–2)	< 0.001

Data are shown as the median with interquartile range or frequency with %. *cTnI* cardiac troponin I, *ULN* upper limit of normal. Positive or negative cTnI were defined as cTnI of ≥ × 1 ULN or < × 1 ULN (< 0.040 ng/ml), respectively. Mean arterial pressure was calculated using the following formula: (systolic blood pressure + 2 × diastolic blood pressure)/3).

### Positive or negative cTnI versus the cumulative event rate of cardiac or non-cardiac death

A total of 5472 (14.9%) deaths including 881 (2.4%) cardiovascular and 4591 (12.5%) non-cardiovascular death occurred within the 360 days of follow-up period. There was more all-cause, cardiovascular, and non-cardiovascular death in patients with positive cTnI compared to patients with negative cTnI (Supplementary Table 1).

**Figure 2:**
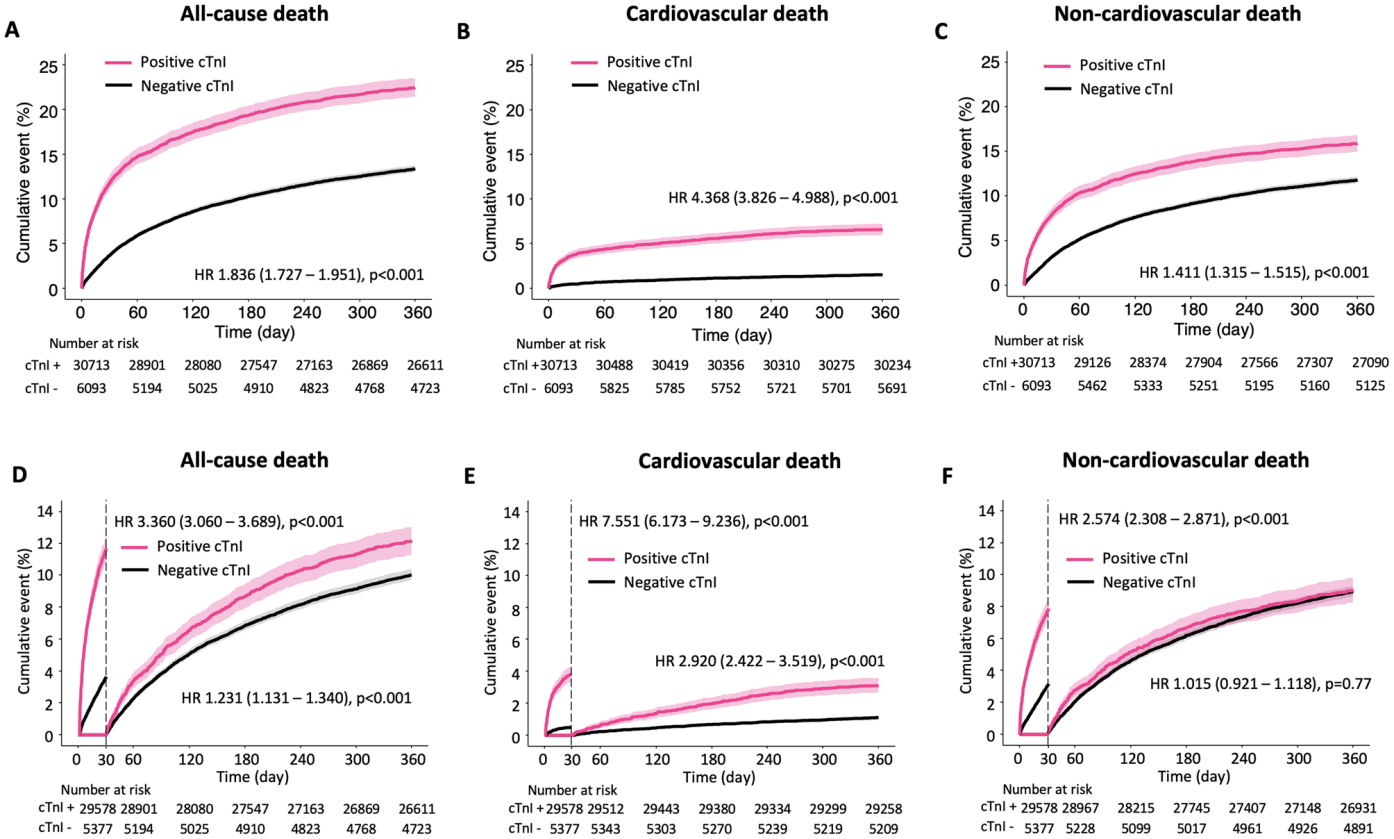
Cumulative event rates of all-cause, cardiovascular, and non-cardiovascular deaths according to positive or negative cTnI levels. The median (interquartile range) and mean ± standard deviation of the follow-up duration was 360 (360–360) days and 321 ± 101 days, respectively. cTnI+ and cTnI− indicate positive cTnI and negative cTnI, respectively. (**A**–**C**) Cumulative event rates of all-cause, cardiovascular, and non-cardiovascular death were 1.8-, 4.4-, and 1.4- fold higher in positive cTnI than in negative cTnI, respectively. (**D**–**F**) In the landmark analysis at day 30, cumulative event rates of all-cause and cardiovascular were 1.2- and 2.9-fold higher in positive cTnI than in negative cTnI, respectively. After day 30, there was no difference in the cumulative event rates of non-cardiovascular death.

The cumulative incidence rates of all-cause, cardiovascular, and non-cardiovascular death were higher in positive cTnI compared to negative cTnI (HR 1.84, 95% CI 1.73–1.95; HR 4.37, 95% CI 3.83–4.99; HR 1.41, 95% CI 1.32–1.52; p < 0.001, all). In the landmark analysis on day 30, the risk of all-cause and cardiovascular death (HR 1.23, 95% CI 1.13–1.34; HR 2.92, 95% CI 2.42–3.52, p < 0.001, all) were higher but the risk of non-cardiovascular death was not higher in positive cTnI compared to negative cTnI (HR 1.02, 95% CI 0.92–1.12, p = 0.77) (Fig. 2).

### Positive or negative cTnI versus the time-dependent risk of cardiovascular or non-cardiovascular death

In the multivariate analysis, a higher hazard of death was evident in the early period for positive cTnI compared to negative cTnI. The hazard of cardiovascular death was > 1 over 360 days, whereas the hazard of non-cardiovascular death became < 1 after approximately 70 days. Consequently, the hazard of all-cause death became non-significant approximately after day 40 (Supplementary Figure 1). The unadjusted analysis and sensitivity analysis using binary clinical variables showed consistent results (Supplementary Figure 2). The statistical results are presented in Supplementary Table 2.

**Figure 3:**
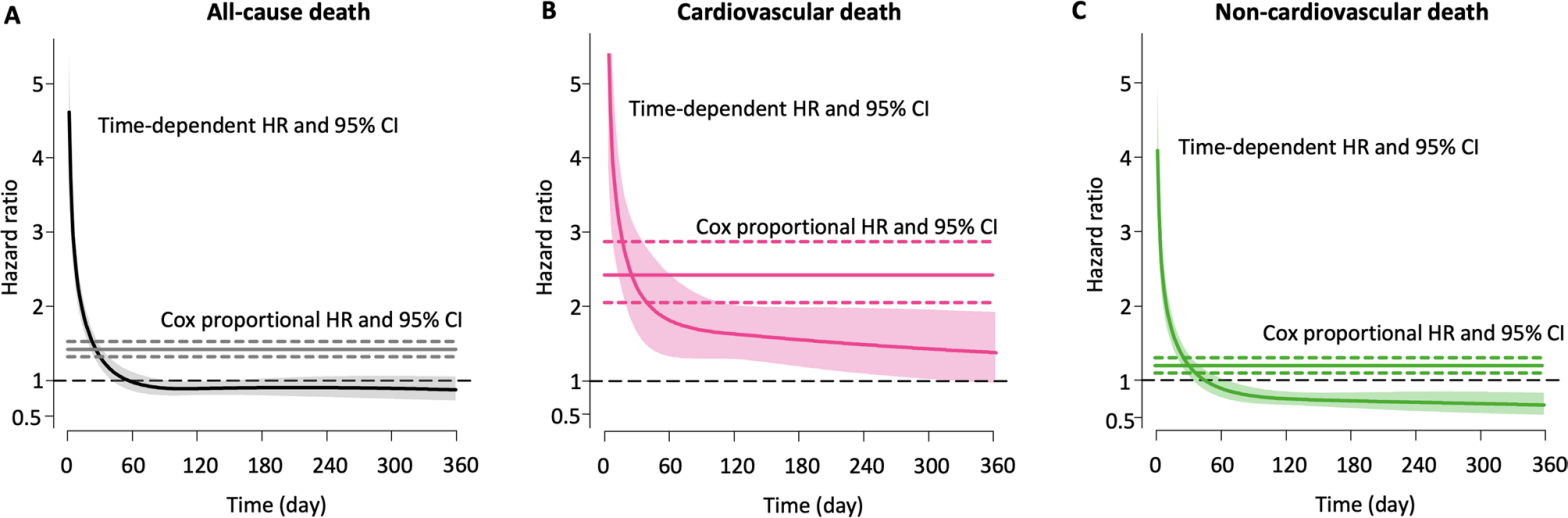
Multivariate-adjusted time-dependent hazard ratios of all-cause-, cardiovascular -, and non-cardiovascular death. Multivariate-adjusted time-dependent hazard ratio (HR) using a flexible parametric survival model are shown. HR and 95% confidence intervals (CI) are shown using spline curves and shaded areas, respectively. For the comparisons, Cox models are also plotted as solid (HR) and dotted (95% CI) horizontal lines. The statistical results are presented in Supplementary Table 1.

In the competing risk analysis using Fine and Grey’s subdistribution hazard model, positive cTnI contributed to the 4.4- and 1.4-fold increased risk of the unadjusted cumulative incidence of cardiovascular and non-cardiovascular death (HR 4.37, 95% CI 3.83–4.99; HR 1.41, 95% CI 1.31–1.52, p < 0.001, all) (Fig. 3). Following the multivariate adjustment, positive cTnI still contributed to the 2.4- and 1.2-fold increased risk of cardiovascular and non-cardiovascular death (HR 2.43, 95% CI 2.04–2.89; HR 1.20, 95% CI 1.09–1.32, p < 0.001, all). A sensitivity analysis using binary clinical variables showed consistent results. Statistical results are presented in Supplementary Table 3.

### cTnI levels and the risk of cardiovascular or non-cardiovascular death

**Figure 4:**
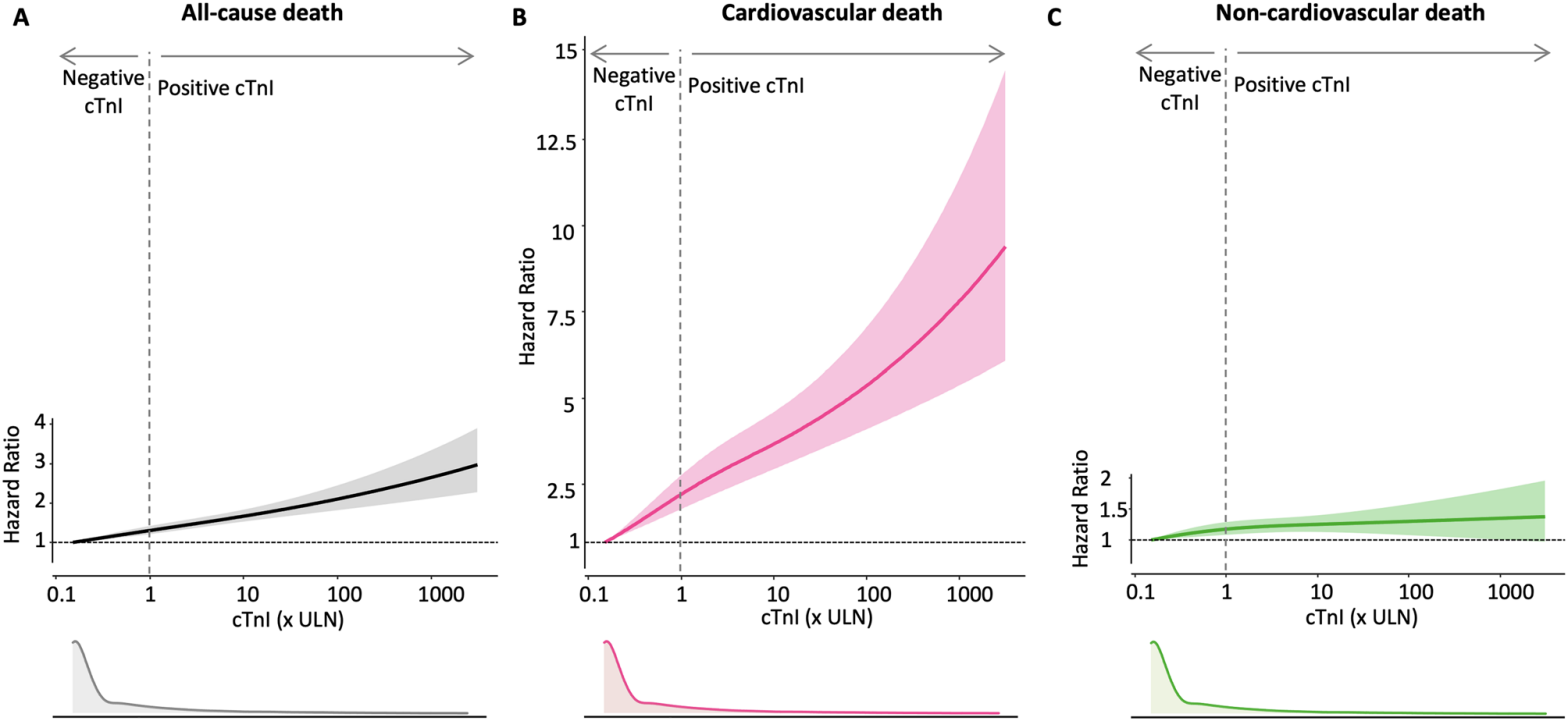
Multivariate-adjusted association between cTnI level and the hazard ratio of all-cause, cardiovascular, or non-cardiovascular death. In the multivariate restricted penalized spline model using variables selected by the Bayesian information criterion (BIC), the risk of all-cause, cardiovascular, and non-cardiovascular death showed a positive relationship to cTnI level. HR and 95% CI are shown using spline curves and shaded areas, respectively. The reference for HR was set to be 1.0 at the lowest cTnI level. The distribution of cTnI was plotted below the x-axis.

The cause-specific death risk was assessed against cTnI levels to investigate whether the death risk increases according to cTnI levels. The risk of all-cause, cardiovascular, and non-cardiovascular death showed positive relationship to cTnI levels in multivariate restricted penalized spline model (Fig. 4). In the sensitivity analysis using binary clinical variables, the risk of all-cause and cardiovascular death showed a positive relationship, and the risk of non-cardiovascular death showed an inverted-U relationship with the cTnI level (Supplementary Figure 3).

## Discussion

This study investigated a large number of unselected patients visiting the emergency department, and showed association among the cTnI level, time, and cause-specific death risk. Positive cTnI was associated with a 2.4-fold higher risk of cardiovascular death and 1.2-fold higher non-cardiovascular death by competing risk analysis. This excess risk of death was mostly evident in the early period and was attenuated thereafter. Both cardiovascular and non-cardiovascular death risk was proportional to cTnI levels.

The results of our study can be summarized in two clinical messages. First, the troponin test should be interpreted as a spectrum of risk, not a binary positive or negative result based on the diagnostic ULN cutoff. Second, the risk diminishes over time and is limited for a few weeks, especially for non-cardiovascular death.

### Interpretation of troponin in clinical practice

In real-world practice, troponin test is frequently performed in patients visiting the emergency department not only for chest pain but also for non-cardiac presentations. Patients often have multiple diseases other than coronary artery disease such as sepsis, pneumonia, renal failure, or cancer. A dichotomous positive or negative report may be too simple to interpret troponin tests^11–14^. The temporal magnitude of cardiovascular or non-cardiovascular death risk provided by troponin test may help physicians to prioritize the clinical flow of each patient, decide whether the patient should be focused on treating the primary disease.

Our results suggest the following clinical scenarios. Any patients with cTnI levels higher than the lowest detectable level might have an increased risk and require preventive measures including sophisticated cardiac testing, risk factor control, and life-style modification. In sicker patients with primary non-cardiovascular disease, and since the long-term outcome would depend on the primary diagnosis, it would be desirable to focus on the primary diagnosis of the patients without seriously pursuing the clinical pathway of acute coronary syndrome. In patients with primary cardiovascular disease, as the excess risk is persistent for at least for 360 days and is mostly evident in the early period, active treatment may be a better approach than conservative waiting. However, further prospective validation in clinical studies would be required to apply above scenarios in clinical practice.

### The hazard of troponin levels lower than ULN

Troponin levels less than the ULN has been used as a reference for the diagnosis of myocardial infarction or prognostic measurement^12,15,16^. Advances in the analytical sensitivity of troponin test have allowed the detection of smaller myocardial infarction. However, it also led to more ambiguity in clinical practice. It is known that the use of the lowest limit of detection showed comparable or better diagnostic sensitivity as well as the cutoff values determined by the 99th percentile of ULN^17–20^. Therefore, complementary use of the lowest limit of detection in ruling-out MI would be helpful for the clinical decisions of emergency department physicians by sorting out patients with low-risk in real-world clinical practice.

In this study, the excess risk of all-cause death and cardiovascular death increased consistently according to the magnitude of cTnI, even in the range below the ULN. Therefore, the clinical implication of troponin may not simply indicate positive or negative risk, rather it might be considered as a continuum of risk. This study also showed that a very small increase of cTnI in the range below the ULN was associated with an increased risk of both cardiovascular and non-cardiovascular death. These results are in line with prior studies that showed association between troponin levels and increased mortality in a variety of clinical settings, including symptomatic or asymptomatic patients^6,21,22^.

### The magnitude of troponin levels and the risk of cardiac or non-cardiac death

This study showed a direct positive relationship between multivariate-adjusted cTnI levels and an excess risk of all-cause death (Fig. 4), which is in line with the previous studies^12,16,21,23,24^. In addition, this study revealed that not only the risk of cardiovascular death but also the risk of non-cardiovascular death was proportional to cTnI levels. This small but definite increase in non-cardiovascular risk at low cTnI levels might be explained by undetected myocardial ischemia, reversible myocardial injury or myocardial strain caused by pressure- or volume-overload, beta-adrenergic stimulation, or impaired troponin clearance in sicker patients^25^. In higher cTnI levels, the long-lasting competing risk of cardiovascular death might mask the increasing risk of non-cardiovascular death, which was unmasked in the competing risk analysis (Supplementary Figure 3).

### Potential bias on the result of cTnI

A single cutoff of cTnI was used for both men and women. Sex‐specific differences in the cardiac troponins are well-recognized, but the clinical implication of using sex‐specific cutoffs compared with a single cutoff remains still unclear^26^. Current European guidelines endorse applying the same high-sensitivity cTnI assay-specific cutoff levels irrespective of age and renal function^27^. However, precise quantification of cTnI might be impaired according to specimen type, hemolysis, hyperbilirubinemia, and presence of interfering heterophile antibodies^28^. Although intact cTnI does not undergo glomerular filtration^29^, cTnI level may falsely increase in renal dysfunction due to the impaired glomerular filtration of smaller cTnI fragments^30^.

### Limitation

This study reports the results of a large dataset derived from real-world clinical practice, and has the inherent limitations of retrospective studies based on electronic medical records. The reported results reflect clinical practice at a tertiary hospital with emergency department overcrowding. Therefore, our results may not be applicable to the other healthcare systems with different clinical situations. cTnI was tested using ADVIA Centaur XP analyzer throughout the study period^31^. The diagnostic capability was quite close to but did not fulfill the criteria for high-sensitivity cardiac troponin, which prevented analysis using the ESC 0/1 rule-out and rule-in algorithm^7,27^.

In the emergency department, the results of initial tests often direct the clinical flow of diagnoses and patient treatments. cTnI testing and frequency of testing were determined by clinical needs, such as chest pain or any possibility of myocardial ischemia, rather than a pre-specified protocol. Therefore, the findings in this study may be applicable to clinical subsets of patients who underwent cTnI testing, but not for all patients visiting the emergency department or with chest pain. Serial cTnI evaluation, which is useful for the discrimination of acute cTnI elevation from chronic cTnI elevation and also for the risk assessment, was not routinely performed^32^.

For the outcome measurement, only cardiovascular or non-cardiovascular deaths were assessed. Specific cardiac events, including myocardial infarction, revascularization, or other detailed clinical events, were not assessed. Adjudication of myocardial infarction type was not performed^2,33,34^. Hypertension identified in the emergency department might be a significant risk factor for incident cardiac or vascular disease, which requires further investigation^35,36^. Family history of coronary artery disease has not been investigated^37,38^.

## Conclusions

Our study showed association between cTnI and the temporal risk distribution of cardiovascular or non-cardiovascular death. In patients visiting the emergency department, cTnI levels higher than the lowest level was associated with an increased risk of both cardiovascular and non-cardiovascular death, which are mostly evident in the early period, especially for non-cardiovascular death. Our results may be used to guide the priority of clinical practice in patients undergoing cTnI tests.

## Supplementary Information

Supplementary Information.
