# Supplementary material for: Cardiac troponin I and the risk of cardiovascular or non-cardiovascular death in patients visiting the emergency department
Source: Sci Rep. 2021 Aug 31;11:17461. doi: 10.1038/s41598-021-96951-y (PMC8408270; doi:10.1038/s41598-021-96951-y)
Supplement: Supplementary file 1 — Supplementary Information. [file 41598_2021_96951_MOESM1_ESM.pdf]

Supplementary Data

Supplementary Table 1. Clinical outcome in 360-day follow-up period

	Negative cTnI (N = 30,713)	Positive cTnI (N = 6,093)	p-value
All-cause death	4102 (13.4)	1370 (22.5)	<0.001
Cardiovascular death	479 (1.6)	402 (6.6)	<0.001
Non-cardiovascular death	3623 (11.8)	968 (15.9)	<0.001
Cause of death			
Cardiovascular disease	479 (11.7)	402 (29.3)	
Cancer	65 (1.6)	35 (2.6)	
Respiratory disease	40 (1.0)	22 (1.6)	
Gastrointestinal disease	138 (3.4)	114 (8.3)	<0.001
Infection	3317 (80.9)	751 (54.8)	
Neurologic disease	9 (0.2)	7 (0.5)	
Trauma	28 (0.7)	21 (1.5)	
Others	26 (0.6)	18 (1.3)	

Supplementary Table 2. Positive cTnI and the risk of all-cause, cardiovascular, and non-cardiovascular death: Generalized survival models for correlated time-to-event data and Cox proportional hazard model

Table 2-A. Unadjusted model

All-cause death

	Time-dependent hazard ratio using flexible parametric survival models		Cox proportional hazard model	
	HR (95% CI)	p-value	HR (95% CI)	p-value
Positive cTnI	19.345 (18.333 - 20.364)	<0.001	1.836 (1.727 - 1.951)	<0.001

Cardiovascular death

	Time-dependent hazard ratio using flexible parametric survival models		Cox proportional hazard model	
	HR (95% CI)	p-value	HR (95% CI)	p-value
Positive cTnI	12.475 (11.297 - 13.667)	<0.001	4.368 (3.826 - 4.988)	<0.001

Non-cardiovascular death

	Time-dependent hazard ratio using flexible parametric survival models		Cox proportional hazard model	
	HR (95% CI)	p-value	HR (95% CI)	p-value
Positive cTnI	32.272 (30.271 - 34.284)	<0.001	1.411 (1.315 - 1.515)	<0.001

Table 2-B. Variables selected by Bayesian information criterion

	All-cause death			
	Time-dependent hazard ratio using flexible parametric survival models		Cox proportional hazard model	
	HR (95% CI)	p-value	HR (95% CI)	p-value
Positive cTnI	15.258 (14.382 - 16.114)	<0.001	1.424 (1.323 - 1.533)	<0.001
Visiting year	0.910 (0.910 - 0.910)	<0.001	0.910 (0.894 - 0.926)	<0.001
Age (years)	1.013 (1.012 - 1.013)	<0.001	1.013 (1.010 - 1.015)	<0.001
Sex	1.358 (1.309 - 1.408)	<0.001	1.350 (1.268 - 1.439)	<0.001
Hypertension	0.791 (0.748 - 0.837)	<0.001	0.792 (0.738 - 0.850)	<0.001
Chronic kidney disease	0.798 (0.698 - 0.908)	0.002	0.791 (0.687 - 0.911)	0.001
History of stroke	0.548 (0.428 - 0.689)	<0.001	0.547 (0.428 - 0.700)	<0.001
History of coronary artery disease	0.474 (0.292 - 0.720)	0.001	0.467 (0.295 - 0.738)	0.001
Respiratory disease	0.825 (0.747 - 0.907)	<0.001	0.824 (0.743 - 0.914)	<0.001
Hepatic disease	1.285 (1.172 - 1.404)	<0.001	1.285 (1.165 - 1.417)	<0.001
Cancer	5.693 (5.498 - 5.891)	<0.001	5.725 (5.328 - 6.153)	<0.001
Endotracheal intubation	2.164 (1.927 - 2.421)	<0.001	2.190 (1.932 - 2.483)	<0.001
Chest pain	0.531 (0.457 - 0.612)	<0.001	0.529 (0.453 - 0.618)	<0.001
Systolic blood pressure (mmHg)	0.993 (0.992 - 0.993)	<0.001	0.993 (0.991 - 0.994)	<0.001
Diastolic blood pressure (mmHg)	1.007 (1.007 - 1.008)	<0.001	1.008 (1.004 - 1.011)	<0.001
Heart rate (/min)	1.008 (1.007 - 1.008)	<0.001	1.008 (1.006 - 1.009)	<0.001
Hemoglobin (g/dL)	0.854 (0.852 - 0.856)	<0.001	0.855 (0.844 - 0.866)	<0.001
White blood cell (103/mm3)	1.007 (1.006 - 1.008)	<0.001	1.007 (1.006 - 1.008)	<0.001
c-reactive protein (mg/dL)	1.021 (1.019 - 1.023)	<0.001	1.021 (1.018 - 1.024)	<0.001
Frequency of cTnI test within 24 hr	0.887 (0.868 - 0.906)	<0.001	0.885 (0.842 - 0.929)	<0.001

Cardiovascular death

	Time-dependent hazard ratio using flexible parametric survival models		Cox proportional hazard model	
	HR (95% CI)	p-value	HR (95% CI)	p-value
Positive cTnI	14.050 (12.575 - 15.526)	0.003	2.430 (2.057 - 2.870)	<0.001
Visiting year	0.922 (0.922 - 0.922)	<0.001	0.922 (0.882 - 0.964)	<0.001
Age (years)	1.025 (1.024 - 1.026)	<0.001	1.025 (1.019 - 1.031)	<0.001
Sex	1.205 (1.093 - 1.324)	0.020	1.204 (1.030 - 1.409)	0.020
Hypertension	0.910 (0.798 - 1.031)	0.29	0.910 (0.763 - 1.084)	0.29
Chronic kidney disease	1.336 (1.049 - 1.671)	0.033	1.338 (1.026 - 1.745)	0.032
History of stroke	1.247 (0.874 - 1.715)	0.24	1.252 (0.869 - 1.803)	0.23
History of coronary artery disease	0.462 (0.198 - 0.893)	0.048	0.460 (0.214 - 0.991)	0.047
Respiratory disease	1.001 (0.783 - 1.256)	0.99	1.002 (0.778 - 1.291)	0.99
Hepatic disease	0.863 (0.617 - 1.168)	0.39	0.864 (0.619 - 1.206)	0.39
Cancer	1.099 (0.971 - 1.238)	0.28	1.100 (0.927 - 1.304)	0.28
Endotracheal intubation	4.312 (3.613 - 5.096)	<0.001	4.341 (3.519 - 5.355)	<0.001
Chest pain	0.784 (0.581 - 1.030)	0.13	0.784 (0.571 - 1.078)	0.13
Systolic blood pressure (mmHg)	0.995 (0.995 - 0.996)	0.024	0.995 (0.991 - 0.999)	0.024
Diastolic blood pressure (mmHg)	0.998 (0.997 - 0.999)	0.66	0.998 (0.991 - 1.006)	0.66
Heart rate (/min)	1.006 (1.005 - 1.007)	<0.001	1.006 (1.003 - 1.009)	<0.001
Hemoglobin (g/dL)	0.866 (0.860 - 0.872)	<0.001	0.866 (0.839 - 0.895)	<0.001
White blood cell (103/mm3)	1.009 (1.008 - 1.010)	<0.001	1.009 (1.008 - 1.011)	<0.001
c-reactive protein (mg/dL)	1.013 (1.007 - 1.019)	0.002	1.013 (1.005 - 1.022)	0.002
Frequency of cTnI test within 24 hr	0.858 (0.815 - 0.901)	0.004	0.856 (0.770 - 0.952)	0.004

Non-cardiovascular death

	Time-dependent hazard ratio using flexible parametric survival models		Cox proportional hazard model	
Parameter	HR (95% CI)	p-value	HR (95% CI)	p-value
Positive cTnI	20.410 (19.038 - 21.741)	<0.001	1.198 (1.101 - 1.303)	<0.001
Visiting year	0.910 (0.910 - 0.910)	<0.001	0.911 (0.894 - 0.928)	<0.001
Age (years)	1.010 (1.010 - 1.011)	<0.001	1.010 (1.008 - 1.013)	<0.001
Sex	1.377 (1.324 - 1.433)	<0.001	1.370 (1.278 - 1.469)	<0.001
Hypertension	0.770 (0.723 - 0.819)	<0.001	0.769 (0.712 - 0.831)	<0.001
Chronic kidney disease	0.686 (0.582 - 0.801)	<0.001	0.681 (0.575 - 0.805)	<0.001
History of stroke	0.358 (0.251 - 0.493)	<0.001	0.357 (0.254 - 0.503)	<0.001
History of coronary artery disease	0.450 (0.241 - 0.755)	0.006	0.445 (0.251 - 0.790)	0.006
Respiratory disease	0.808 (0.725 - 0.896)	<0.001	0.807 (0.721 - 0.905)	<0.001
Hepatic disease	1.327 (1.206 - 1.456)	<0.001	1.327 (1.197 - 1.470)	<0.001
Cancer	7.926 (7.644 - 8.215)	<0.001	7.959 (7.313 - 8.663)	<0.001
Endotracheal intubation	1.242 (1.062 - 1.442)	0.009	1.230 (1.044 - 1.450)	0.013
Chest pain	0.483 (0.405 - 0.570)	<0.001	0.481 (0.402 - 0.576)	<0.001
Systolic blood pressure (mmHg)	0.992 (0.992 - 0.992)	<0.001	0.992 (0.990 - 0.994)	<0.001
Diastolic blood pressure (mmHg)	1.010 (1.009 - 1.010)	<0.001	1.010 (1.007 - 1.013)	<0.001
Heart rate (/min)	1.007 (1.007 - 1.008)	<0.001	1.007 (1.006 - 1.009)	<0.001
Hemoglobin (g/dL)	0.861 (0.859 - 0.863)	<0.001	0.862 (0.850 - 0.875)	<0.001
White blood cell (103/mm3)	1.004 (1.002 - 1.005)	<0.001	1.004 (1.002 - 1.005)	<0.001
c-reactive protein (mg/dL)	1.021 (1.019 - 1.024)	<0.001	1.021 (1.018 - 1.025)	<0.001
Frequency of cTnI test within 24 hr	0.905 (0.883 - 0.927)	<0.001	0.904 (0.856 - 0.956)	<0.001

Table 2-C. Binary clinical parameters

All-cause death

	Time-dependent hazard ratio using flexible parametric survival models		Cox proportional hazard model	
Parameter	HR (95% CI)	p-value	HR (95% CI)	p-value
Positive cTnI	13.731 (13.010 - 14.441)	<0.001	1.533 (1.434 - 1.639)	<0.001
Age ≥ 65 year	1.271 (1.225 - 1.319)	<0.001	1.272 (1.202 - 1.345)	<0.001
Sex	1.338 (1.294 - 1.383)	<0.001	1.334 (1.261 - 1.412)	<0.001
Smoking	0.846 (0.754 - 0.945)	0.006	0.842 (0.748 - 0.948)	0.005
Diabetes	0.922 (0.850 - 0.998)	0.08	0.916 (0.837 - 1.004)	0.06
Hypertension	0.803 (0.763 - 0.844)	<0.001	0.803 (0.752 - 0.858)	<0.001
Chronic kidney disease	0.945 (0.836 - 1.062)	0.38	0.934 (0.822 - 1.061)	0.30
History of stroke	0.655 (0.526 - 0.803)	<0.001	0.656 (0.527 - 0.815)	<0.001
History of coronary artery disease	0.369 (0.230 - 0.554)	0.001	0.367 (0.202 - 0.666)	0.001
History of myocardial infarction	1.580 (0.758 - 2.844)	0.31	1.555 (0.644 - 3.754)	0.33
Respiratory disease	0.838 (0.767 - 0.914)	<0.001	0.842 (0.766 - 0.924)	<0.001
Hepatic disease	1.327 (1.224 - 1.437)	<0.001	1.327 (1.217 - 1.448)	<0.001
Cancer	7.715 (7.477 - 7.958)	<0.001	7.745 (7.278 - 8.243)	<0.001
Endotracheal intubation	2.153 (1.943 - 2.377)	<0.001	2.177 (1.934 - 2.451)	<0.001
Use of vasopressor	1.401 (1.310 - 1.497)	<0.001	1.403 (1.282 - 1.537)	<0.001
Chest pain	0.392 (0.341 - 0.447)	<0.001	0.390 (0.339 - 0.449)	<0.001
Mean arterial pressure < 70 mmHg	1.277 (1.189 - 1.369)	<0.001	1.274 (1.172 - 1.386)	<0.001
Heart rate ≥ 100/min	1.789 (1.722 - 1.857)	<0.001	1.783 (1.686 - 1.885)	<0.001

Cardiovascular death

Parameter	Time-dependent hazard ratio using flexible parametric survival models		Cox proportional hazard model	
	HR (95% CI)	p-value	HR (95% CI)	p-value
Positive cTnI	11.539 (10.446 - 12.642)	0.001	2.498 (2.153 - 2.898)	<0.001
Age ≥ 65 year	2.249 (2.072 - 2.436)	<0.001	2.249 (1.940 - 2.608)	<0.001
Sex	1.130 (1.036 - 1.229)	0.08	1.130 (0.983 - 1.298)	0.08
Smoking	0.737 (0.553 - 0.958)	0.040	0.735 (0.549 - 0.984)	0.039
Diabetes	1.145 (0.970 - 1.339)	0.18	1.145 (0.938 - 1.397)	0.18
Hypertension	0.822 (0.731 - 0.920)	0.018	0.822 (0.698 - 0.967)	0.018
Chronic kidney disease	1.710 (1.387 - 2.080)	<0.001	1.715 (1.361 - 2.161)	<0.001
History of stroke	1.370 (0.996 - 1.827)	0.06	1.380 (0.996 - 1.911)	0.05
History of coronary artery disease	0.178 (0.081 - 0.329)	0.015	0.177 (0.044 - 0.716)	0.015
History of myocardial infarction	5.546 (2.197 - 11.199)	0.036	5.553 (1.120 - 27.542)	0.036
Respiratory disease	0.993 (0.799 - 1.217)	0.95	0.993 (0.793 - 1.245)	0.95
Hepatic disease	1.061 (0.811 - 1.358)	0.67	1.064 (0.810 - 1.397)	0.66
Cancer	1.450 (1.301 - 1.608)	<0.001	1.450 (1.255 - 1.675)	<0.001
Endotracheal intubation	3.043 (2.598 - 3.536)	<0.001	3.062 (2.478 - 3.782)	<0.001
Use of vasopressor	2.292 (2.023 - 2.583)	<0.001	2.294 (1.884 - 2.794)	<0.001
Chest pain	0.560 (0.423 - 0.722)	<0.001	0.559 (0.420 - 0.745)	<0.001
Mean arterial pressure < 70 mmHg	1.207 (1.025 - 1.409)	0.06	1.206 (0.994 - 1.465)	0.06
Heart rate ≥ 100/min	1.424 (1.290 - 1.568)	<0.001	1.424 (1.233 - 1.645)	<0.001

Non-cardiovascular death

Parameter	Time-dependent hazard ratio using flexible parametric survival models HR (95% CI)	p-value	Cox proportional hazard model HR (95% CI)	p-value
Positive cTnI	18.849 (17.679 - 19.994)	<0.001	1.295 (1.200 - 1.398)	<0.001
Age ≥ 65 year	1.131 (1.085 - 1.179)	<0.001	1.133 (1.065 - 1.204)	<0.001
Sex	1.395 (1.346 - 1.446)	<0.001	1.391 (1.307 - 1.480)	<0.001
Smoking	0.875 (0.771 - 0.988)	0.044	0.874 (0.768 - 0.996)	0.043
Diabetes	0.877 (0.798 - 0.960)	0.012	0.873 (0.787 - 0.967)	0.010
Hypertension	0.804 (0.759 - 0.850)	<0.001	0.804 (0.748 - 0.864)	<0.001
Chronic kidney disease	0.765 (0.657 - 0.884)	0.001	0.758 (0.648 - 0.885)	<0.001
History of stroke	0.442 (0.324 - 0.586)	<0.001	0.441 (0.326 - 0.596)	<0.001
History of coronary artery disease	0.455 (0.244 - 0.762)	0.019	0.454 (0.234 - 0.879)	0.019
History of myocardial infarction	0.664 (0.165 - 1.718)	0.54	0.652 (0.177 - 2.411)	0.52
Respiratory disease	0.819 (0.743 - 0.901)	<0.001	0.822 (0.741 - 0.911)	<0.001
Hepatic disease	1.329 (1.220 - 1.445)	<0.001	1.328 (1.212 - 1.456)	<0.001
Cancer	10.867 (10.517 - 11.225)	<0.001	10.888 (10.114 - 11.721)	<0.001
Endotracheal intubation	1.436 (1.253 - 1.636)	<0.001	1.427 (1.226 - 1.661)	<0.001
Use of vasopressor	1.184 (1.092 - 1.281)	0.002	1.182 (1.065 - 1.313)	0.002
Chest pain	0.370 (0.315 - 0.431)	<0.001	0.368 (0.313 - 0.433)	<0.001
Mean arterial pressure < 70 mmHg	1.282 (1.184 - 1.385)	<0.001	1.280 (1.167 - 1.404)	<0.001
Heart rate ≥ 100/min	1.816 (1.743 - 1.891)	<0.001	1.809 (1.703 - 1.922)	<0.001

Supplementary Table 3. Subdistribution hazard models and cause-specific hazard models for cardiovascular and non-cardiovascular death

Table 3-A. Unadjusted model

	**Subdistribution hazard model**			
	Cardiovascular death		Non-cardiovascular death	
	sHR (95% CI)	p-value	sHR (95% CI)	p-value
Positive cTnI	4.370 (3.830 - 4.990)	<0.001	1.410 (1.310 - 1.520)	<0.001

	**Cause-specific hazard model**			
	Cardiovascular death		Non-cardiovascular death	
	HR (95% CI)	p-value	HR (95% CI)	p-value
Positive cTnI	4.571 (4.003 - 5.219)	<0.001	1.471 (1.370 - 1.579)	<0.001

Table 3-B. Variables selected by Bayesian information criterion

Subdistribution hazard model

	Cardiovascular death		Non--cardiovascular death	
	sHR (95% CI)	p-value	sHR (95% CI)	p-value
Visiting year	0.922 (0.883 - 0.963)	<0.001	0.911 (0.894 - 0.928)	<0.001
Positive cTnI	2.430 (2.041 - 2.892)	<0.001	1.198 (1.091 - 1.315)	<0.001
Age (years)	1.025 (1.018 - 1.032)	<0.001	1.010 (1.007 - 1.013)	<0.001
Sex	1.204 (1.023 - 1.418)	0.025	1.370 (1.276 - 1.472)	<0.001
Hypertension	0.910 (0.758 - 1.092)	0.310	0.769 (0.710 - 0.833)	<0.001
Chronic kidney disease	1.338 (1.012 - 1.768)	0.041	0.681 (0.574 - 0.807)	<0.001
History of stroke	1.252 (0.875 - 1.792)	0.220	0.357 (0.255 - 0.501)	<0.001
History of coronary artery disease	0.460 (0.215 - 0.985)	0.046	0.445 (0.253 - 0.783)	0.005
Respiratory disease	1.002 (0.776 - 1.294)	0.990	0.807 (0.714 - 0.913)	0.001
Hepatic disease	0.864 (0.614 - 1.214)	0.400	1.327 (1.189 - 1.480)	<0.001
Cancer	1.100 (0.917 - 1.318)	0.300	7.959 (7.286 - 8.694)	<0.001
Chest pain	0.784 (0.563 - 1.092)	0.150	0.481 (0.403 - 0.574)	<0.001
Endotracheal intubation	4.341 (3.419 - 5.512)	<0.001	1.230 (0.999 - 1.516)	0.051
Systolic blood pressure (mmHg)	0.995 (0.991 - 1.000)	0.036	0.992 (0.990 - 0.994)	<0.001
Diastolic blood pressure (mmHg)	0.998 (0.991 - 1.006)	0.690	1.010 (1.006 - 1.013)	<0.001
Heart rate (/min)	1.006 (1.003 - 1.010)	<0.001	1.007 (1.006 - 1.009)	<0.001
Hemoglobin (g/dL)	0.866 (0.835 - 0.898)	<0.001	0.862 (0.849 - 0.875)	<0.001
White blood cell ($10^3/mm^3$)	1.009 (1.007 - 1.012)	<0.001	1.004 (1.001 - 1.006)	0.001
c-reactive protein (mg/dL)	1.013 (1.004 - 1.022)	<0.001	1.021 (1.017 - 1.025)	<0.001
Frequency of cTnI test within 24 hr	0.856 (0.763 - 0.960)	0.008	0.904 (0.853 - 0.959)	0.001

Cause-specific hazard model

	Cardiovascular death		Non--cardiovascular death	
	HR (95% CI)	p-value	HR (95% CI)	p-value
Visiting year	0.922 (0.882 - 0.964)	<0.001	0.909 (0.892 - 0.927)	<0.001
Positive cTnI	2.430 (2.057 - 2.870)	<0.001	1.254 (1.153 - 1.364)	<0.001
Age (year)	1.025 (1.019 - 1.031)	<0.001	1.010 (1.007 - 1.013)	<0.001
Sex	1.204 (1.030 - 1.409)	0.005	1.379 (1.286 - 1.478)	<0.001
Hypertension	0.910 (0.763 - 1.084)	0.239	0.778 (0.720 - 0.840)	<0.001
Chronic kidney disease	1.338 (1.026 - 1.745)	0.084	0.676 (0.571 - 0.800)	<0.001
History of stroke	1.252 (0.869 - 1.803)	0.385	0.358 (0.254 - 0.505)	<0.001
History of coronary artery disease	0.460 (0.214 - 0.991)	0.043	0.437 (0.246 - 0.776)	0.005
Respiratory disease	1.002 (0.778 - 1.291)	0.814	0.809 (0.722 - 0.907)	<0.001
Hepatic disease	0.864 (0.619 - 1.206)	0.566	1.331 (1.202 - 1.475)	<0.001
Cancer	1.100 (0.927 - 1.304)	<0.001	8.066 (7.409 - 8.781)	<0.001
Chest pain	0.784 (0.571 - 1.078)	0.112	0.484 (0.404 - 0.579)	<0.001
Endotracheal intubation	4.341 (3.519 - 5.355)	<0.001	1.545 (1.313 - 1.820)	<0.001
Systolic blood pressure (mmHg)	0.995 (0.991 - 0.999)	0.008	0.992 (0.990 - 0.994)	<0.001
Diastolic blood pressure (mmHg)	0.998 (0.991 - 1.006)	0.745	1.009 (1.006 - 1.013)	<0.001
Heart rate (/min)	1.006 (1.003 - 1.009)	<0.001	1.008 (1.006 - 1.009)	<0.001
Hemoglobin (g/dL)	0.866 (0.839 - 0.895)	<0.001	0.859 (0.846 - 0.871)	<0.001
White blood cell (10^3/mm^3)	1.009 (1.008 - 1.011)	<0.001	1.005 (1.004 - 1.007)	<0.001
c-reactive protein (mg/dL)	1.013 (1.005 - 1.022)	<0.001	1.022 (1.019 - 1.026)	<0.001
Frequency of cTnI test within 24 hr	0.856 (0.770 - 0.952)	0.002	0.897 (0.848 - 0.948)	<0.001

Table 3-C. Binary clinical parameters

Subdistribution hazard model

	Cardiovascular death		Non-–cardiovascular death	
	sHR (95% CI)	p-value	sHR (95% CI)	p-value
Positive cTnI	2.498 (2.133 - 2.925)	<0.001	1.295 (1.192 - 1.407)	<0.001
Age ≥ 65 year	2.249 (1.933 - 2.618)	<0.001	1.133 (1.062 - 1.207)	<0.001
Sex	1.130 (0.979 - 1.304)	0.094	1.391 (1.306 - 1.481)	<0.001
Smoking	0.735 (0.546 - 0.989)	0.042	0.874 (0.765 - 0.999)	0.049
Diabetes	1.145 (0.937 - 1.399)	0.190	0.873 (0.785 - 0.970)	0.012
Hypertension	0.822 (0.696 - 0.969)	0.020	0.803 (0.747 - 0.865)	<0.001
Chronic kidney disease	1.715 (1.356 - 2.168)	0.000	0.758 (0.648 - 0.886)	0.001
Respiratory disease	0.993 (0.790 - 1.249)	0.950	0.822 (0.739 - 0.914)	<0.001
Hepatic disease	1.064 (0.806 - 1.404)	0.660	1.328 (1.206 - 1.463)	<0.001
History of stroke	1.380 (0.995 - 1.913)	0.053	0.441 (0.324 - 0.599)	<0.001
History of coronary artery disease	0.177 (0.045 - 0.698)	0.013	0.454 (0.235 - 0.877)	0.019
History of myocardial infarction	5.549 (1.133 -27.192)	0.035	0.652 (0.177 - 2.398)	0.520
Cancer	1.450 (1.245 - 1.689)	<0.001	10.888 (10.090 - 1.749)	<0.001
Endotracheal intubation	3.062 (2.443 - 3.837)	<0.001	1.427 (1.194 - 1.705)	<0.001
Use of vasopressor	2.294 (1.855 - 2.838)	<0.001	1.182 (1.055 - 1.324)	0.004
Chest pain	0.559 (0.417 - 0.750)	<0.001	0.368 (0.315 - 0.431)	<0.001
Mean arterial pressure < 70 mmHg	1.206 (0.981 - 1.484)	0.076	1.280 (1.158 - 1.414)	<0.001
Heart rate ≥ 100/min	1.424 (1.222 - 1.659)	<0.001	1.809 (1.701 - 1.924)	<0.001

Cause-specific hazard model

	Cardiovascular death		Non--cardiovascular death	
	HR (95% CI)	p-value	HR (95% CI)	p-value
Positive cTnI	2.585 (2.226 - 3.002)	<0.001	1.352 (1.253 - 1.459)	<0.001
Age ≥ 65 year	2.224 (1.917 - 2.579)	<0.001	1.146 (1.078 - 1.219)	<0.001
Sex	1.162 (1.011 - 1.335)	0.034	1.390 (1.306 - 1.479)	<0.001
Smoking	0.714 (0.534 - 0.956)	0.024	0.871 (0.765 - 0.991)	0.037
Diabetes	1.126 (0.922 - 1.375)	0.246	0.866 (0.781 - 0.960)	0.006
Hypertension	0.821 (0.697 - 0.966)	0.017	0.807 (0.751 - 0.867)	<0.001
Chronic kidney disease	1.641 (1.301 - 2.068)	<0.001	0.758 (0.649 - 0.886)	0.001
Respiratory disease	0.992 (0.792 - 1.243)	0.945	0.818 (0.738 - 0.907)	<0.001
Hepatic disease	1.120 (0.853 - 1.470)	0.414	1.343 (1.225 - 1.472)	<0.001
History of stroke	1.304 (0.941 - 1.805)	0.110	0.443 (0.328 - 0.598)	<0.001
History of coronary artery disease	0.176 (0.043 - 0.712)	0.015	0.445 (0.230 - 0.862)	0.016
History of myocardial infarction	5.427 (1.094 - 6.920)	0.038	0.652 (0.176 - 2.410)	0.521
Cancer	1.812 (1.569 - 2.093)	<0.001	11.040 (10.255 - 11.885)	<0.001
Endotracheal intubation	3.398 (2.754 - 4.193)	<0.001	1.711 (1.472 - 1.989)	<0.001
Use of vasopressor	2.424 (1.993 - 2.949)	<0.001	1.234 (1.112 - 1.369)	<0.001
Chest pain	0.536 (0.403 - 0.714)	<0.001	0.365 (0.310 - 0.429)	<0.001
Mean arterial pressure < 70 mmHg	1.267 (1.043 - 1.538)	0.017	1.283 (1.169 - 1.407)	<0.001
Heart rate ≥ 100/min	1.521 (1.318 - 1.755)	<0.001	1.830 (1.723 - 1.944)	<0.001

Supplementary Figure 1. Competing risk of cardiovascular or non-cardiovascular death

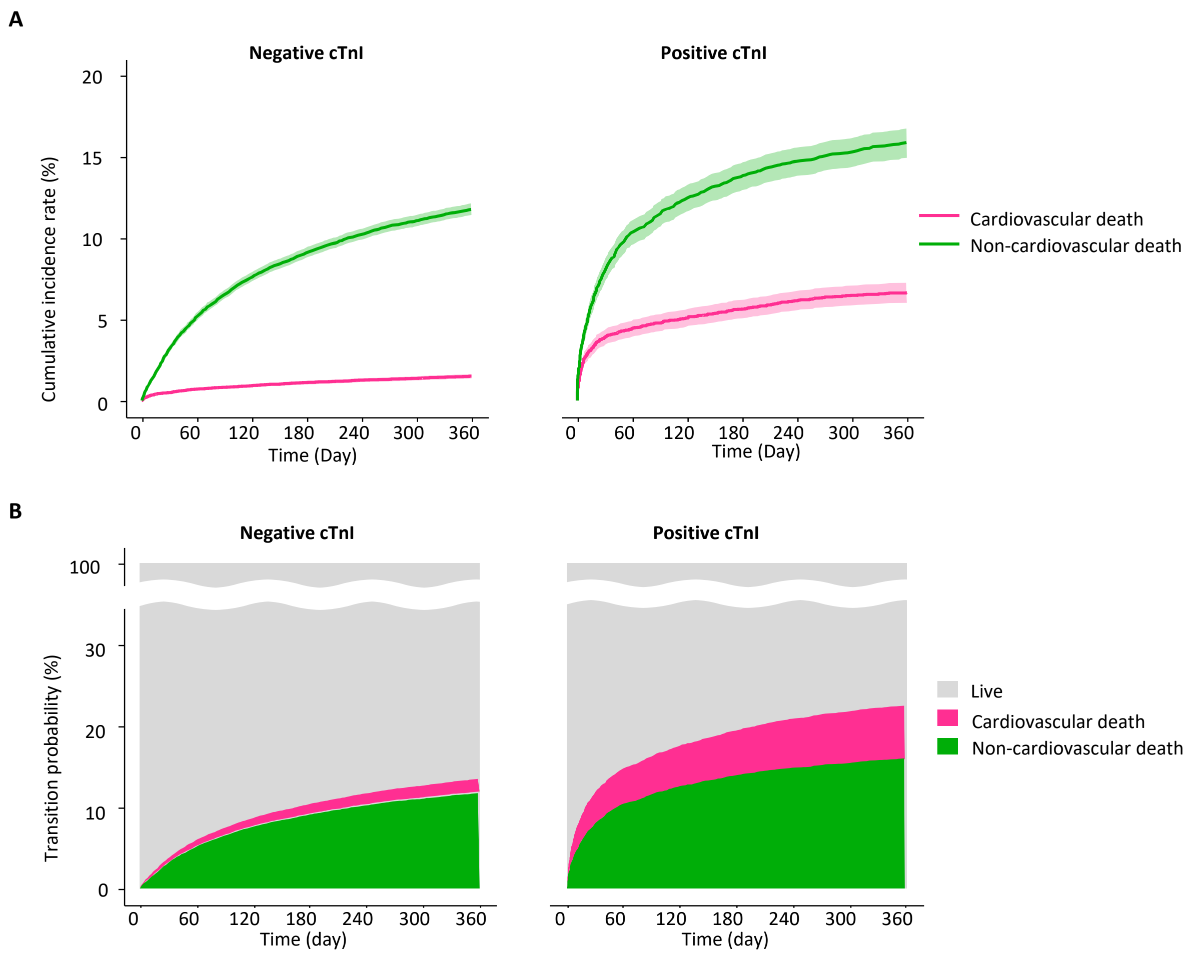

A. Competing risk of cardiovascular or non-cardiovascular death in patients with negative cTnI and positive cTnI, respectively.

B. Stacked transitional probability plots of live, cardiovascular death, or non-cardiovascular death in patients with negative cTnI and positive cTnI, respectively.

Statistical results are presented in Supplementary Table 3.

Supplementary Figure 2. Unadjusted time-dependent hazard ratio using flexible parametric survival models

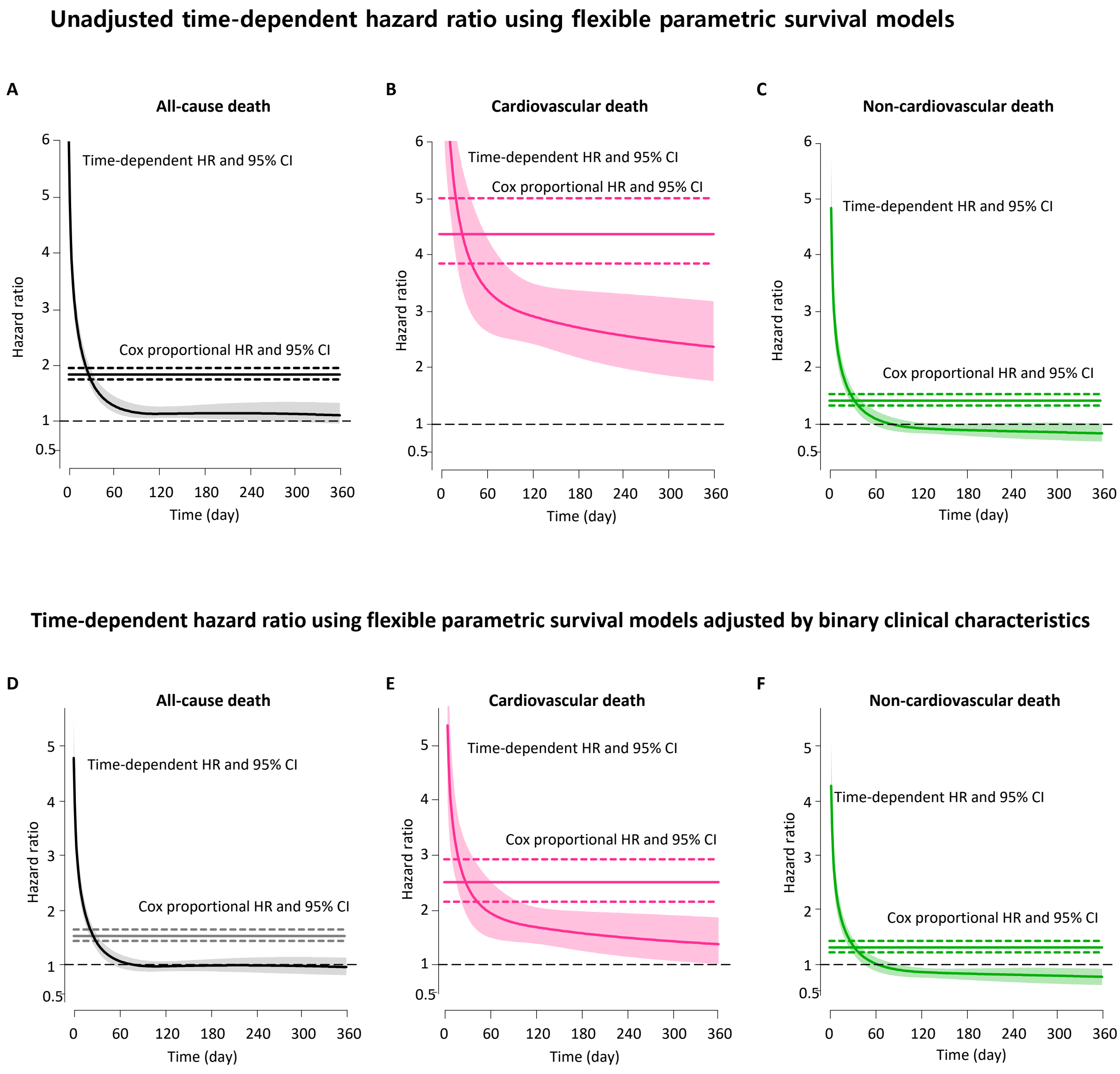

Time-dependent hazard ratio using flexible parametric survival models are shown in thick colored solid lines. For comparison, Cox models are also plotted in grey colored solid (HR) and dotted (95% CI) lines.

Panels A-C: Unadjusted Time-dependent hazard ratio using flexible parametric survival models.

Panels D-F: Spline curves adjusted with the following binary clinical characteristics: visiting year, age, sex, smoking, hypertension, diabetes, chronic kidney disease, lung disease, liver disease, prior history of stroke, prior history of coronary artery disease, prior history of myocardial infarction, cancer, endotracheal intubation, use of inotropic agent, chest pain, dyspnea, hypotension defined by mean arterial pressure < 70 mmHg, and tachycardia defined by ≥ 100/min.

Coefficients of model covariates are presented in Supplementary Table 2.

Supplementary Figure 3. Association between TnI level and the hazard ratio of all-cause, cardiovascular, or non-cardiovascular death

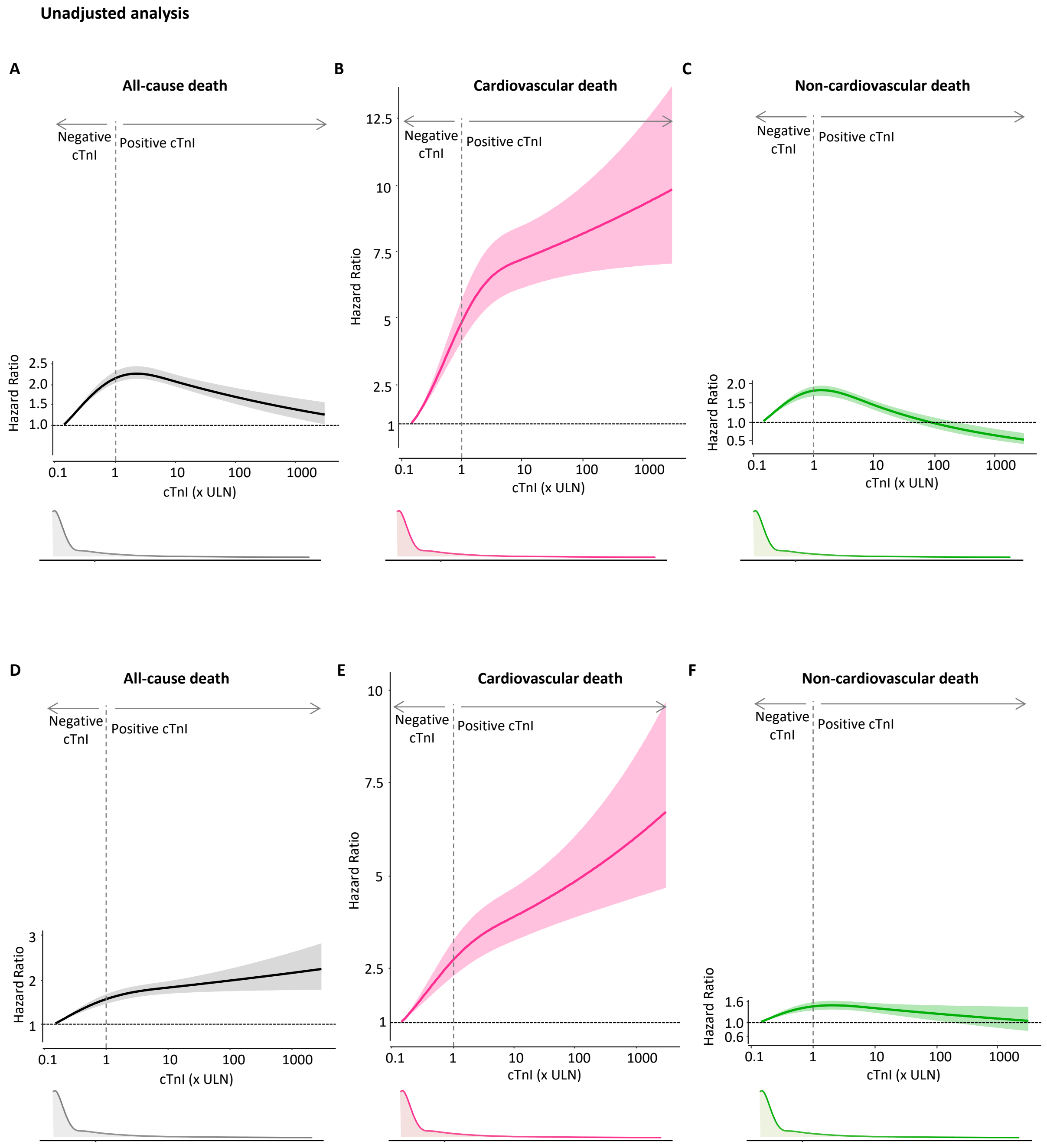

Unadjusted restricted penalized spline model (panels A-C) and multivariate restricted penalized spline model using binary clinical parameters (panels D-F) are shown. The hazard of all-cause, cardiovascular, and non-cardiovascular death showed a positive relationship with

cTnI level. Hazard ratio and 95% confidence intervals are shown using spline curves and shaded areas, respectively. The probability distribution of cTnI is plotted below the x axis. The reference for the hazard ratio was set to be 1.0 at the lowest cTnI level.

Supplementary document - Operational definitions

The clinical characteristics were defined by the following lists. The following diagnostic codes were used for the primary diagnosis. For risk factors such as hypertension or diabetes, secondary diagnoses were also used.

1. International Classification of Disease 10th revision code (ICD-10) or Korea national health insurance code (KNHI code):

- Hypertension (ICD-10 I10.x, I11.x, I12.x, I13.x, I14.x, I15.x)
- Dyslipidemia (ICD-10 E78.x)
- Diabetes mellitus (ICD-10 E10.x, E11.x, E12.x, E13.x, E14.x)
- Chronic kidney disease (ICD-10 N03.2-N03.7, N05.2-N05.7, N19.x, N25.0, Z49.0-Z49.2, Z94.0, Z99.2)
- Coronary artery disease (ICD-10 I20.x, I21.x, I23.x, I24.x, I25.x)
- Congestive heart failure (ICD-10 I09.9, I11.0, I13.0, I13.2, I25.5, I42.0, I42.5-I42.9, I43.x, P29.0)
- Respiratory disease (ICD-10 I27.8, I27.9, J40.x, J67.x, J68.4, J70.1, J70.3)
- Liver disease (ICD-10 B18.x, K70.0-K70.3, K70.9, K71.3-K71.5, K74.0-K74.2, K76.0, K76.2-K76.4, K76.8, K76.9, Z94.4, K70.4, K71.1, K76.5)
- Cancer (Registered in Korean National Health Insurance Service)
- Stroke (ICD-10 I60.x, I61.x, I62.x, I63.x)

2. Administrative codes and data of of Darwin-C data warehouse system

– Age

– Sex

– Timestamp of visiting and discharge of emergency department

– Timestamp of death

– Timestamp and results of laboratory test

– Medications and procedures including use of vasopressor, endotracheal intubation

3. Proprietary text search and processing engines of Darwin-C data warehouse system

– Symptom
